# The Role of Short-Chain Fatty Acids and Bile Acids in Intestinal and Liver Function, Inflammation, and Carcinogenesis

**DOI:** 10.3389/fcell.2021.703218

**Published:** 2021-07-26

**Authors:** Alexander Visekruna, Maik Luu

**Affiliations:** ^1^Institute for Medical Microbiology and Hygiene, Philipps-University Marburg, Marburg, Germany; ^2^Medizinische Klinik und Poliklinik II, Universitätsklinikum Würzburg, Würzburg, Germany

**Keywords:** liver, microbiome and dysbiosis, intestine, immunology, short-chain fatty acid, bile acids, T cell, myeloid cells

## Abstract

During the past decade, researchers have investigated the role of microbiota in health and disease. Recent findings support the hypothesis that commensal bacteria and in particular microbiota-derived metabolites have an impact on development of inflammation and carcinogenesis. Major classes of microbial-derived molecules such as short-chain fatty acids (SCFA) and secondary bile acids (BAs) were shown to have immunomodulatory potential in various autoimmune, inflammatory as well as cancerous disease models and are dependent on diet-derived substrates. The versatile mechanisms underlying both beneficial and detrimental effects of bacterial metabolites comprise diverse regulatory pathways in lymphocytes and non-immune cells including changes in the signaling, metabolic and epigenetic status of these. Consequently, SCFAs as strong modulators of immunometabolism and histone deacetylase (HDAC) inhibitors have been investigated as therapeutic agents attenuating inflammatory and autoimmune disorders. Moreover, BAs were shown to modulate the microbial composition, adaptive and innate immune response. In this review, we will discuss the recent findings in the field of microbiota-derived metabolites, especially with respect to the molecular and cellular mechanisms of SCFA and BA biology in the context of intestinal and liver diseases.

## Introduction

The triangular interdependency between gut microbiota, diet and immune cells is substantially connected to the functionality of a symbiotic cellular network and therefore to the host’s health status. The gut as residence for a highly dense microbial community harbors a unique diversity of non-mammalian genes required for the synthesis of various bioactive molecules. These soluble messengers are bridging the gap between host cells as well as commensal bacteria and are required for the maintenance of energy homeostasis, shaping the mucosal immune system and even influencing host behavior (Blumberg and Powrie, 2012; Levy et al., 2016). Changes in the microbiota have been shown to be involved in pathophysiological processes. While microbial diversity is associated with a beneficial outcome in allogenic stem cell transplantation, the impact of the gut microbiota on checkpoint blockade in cancer therapy showed opposing effects (Coutzac et al., 2020; Peled et al., 2020).

**FIGURE 1:**
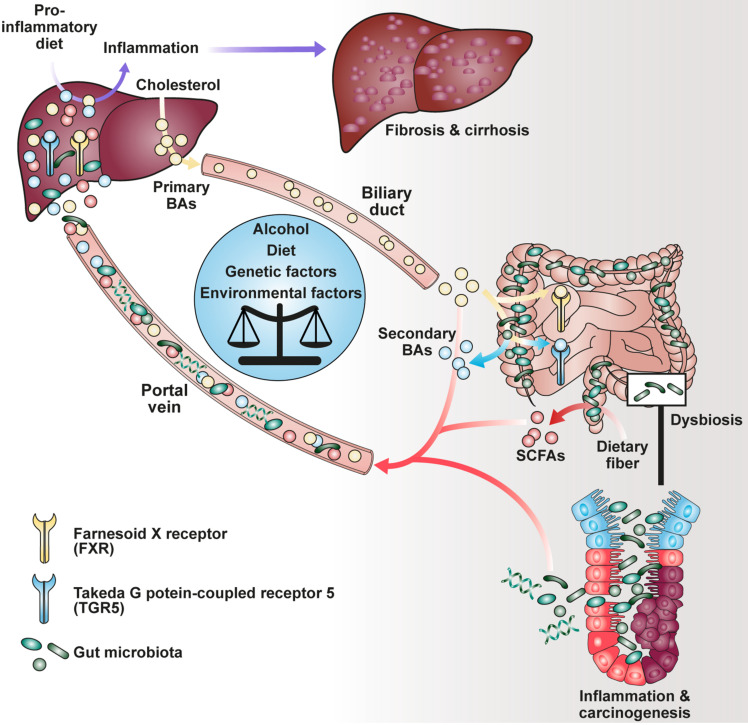
Impact of BAs and SCFAs on the gut-liver-axis.

The production of short-chain fatty acids (SCFAs), a major class of microbial metabolites, requires bacterial fermentation of both water-soluble dietary fiber (e.g., pectin, guar gum, and inulin) and insoluble fiber (e.g., resistant starch) in the gut lumen by members of the human microbiome (Figure 1). Upon food intake, indigestible complex carbohydrates pass the upper part of the gastrointestinal tract, where they become metabolized under anaerobic conditions with peak concentration of SCFAs in the cecum and proximal colon (Cummings et al., 1987). Acetate, propionate and butyrate are the most abundant SCFAs in the gut of conventionally raised mice. Additionally, pentanoate, formate and branched-chain fatty acids (BCFAs) have been identified at much lower levels in the intestine of rodents and humans (Koh et al., 2016). In contrast to SCFAs and lactate, which is another product of the carbohydrate metabolism, BCFAs are derived from fermentation of branched amino acids such as valine, leucine and isoleucine (Smith, 1998; Yang et al., 2010; Rios-Covian et al., 2020). SCFAs are absent in germ-free (GF) animals and were shown to affect different aspects of human health. These implicate, besides autoimmunity and inflammation, the maintenance of gut homeostasis, an equilibrium of interactions between the intestinal epithelium, the host immune system, commensal bacteria and regulatory mechanisms (Kim et al., 2014; Luu et al., 2019).

Notably, besides dietary components, commensal bacteria are able to modify host-derived molecules such as bile acids (BAs). Upon food uptake, stimulation of the gallbladder leads to influx of primary liver-derived BAs into the duodenum being responsible for emulsification of dietary fat (Ridlon et al., 2006). Although most BAs become reabsorbed in the ileum and transported to the liver via the enterohepatic circulation, a smaller fraction is transformed into secondary BAs by bacterial conversion in the colon (Schaap et al., 2014). It has been described that both primary and secondary BAs interact with a family of nuclear (FXR) and G-protein-coupled receptors (GPRs) agonistically or antagonistically, collectively known as BA-activated receptors (BARs), thereby modulating cellular signaling as well as immune response (Chen et al., 2011; Carr and Reid, 2015). Recently, it has been shown that secondary BAs such as 3β-hydroxydeoxycholic acid (isoDCA) were able to increase differentiation of regulatory T cells (Tregs) by interaction with the farnesoid X receptor on dendritic cells (DCs) highlighting a potential for novel therapeutics (Campbell et al., 2020b).

In this review, we examine recent work investigating the modes of action by which two major groups of bacterial metabolites, SCFAs and BAs, impact on liver- and gut-associated inflammatory and cancerous diseases.

## Mechanisms of SCFA-Mediated Modification of Host Cells

**FIGURE 2:**
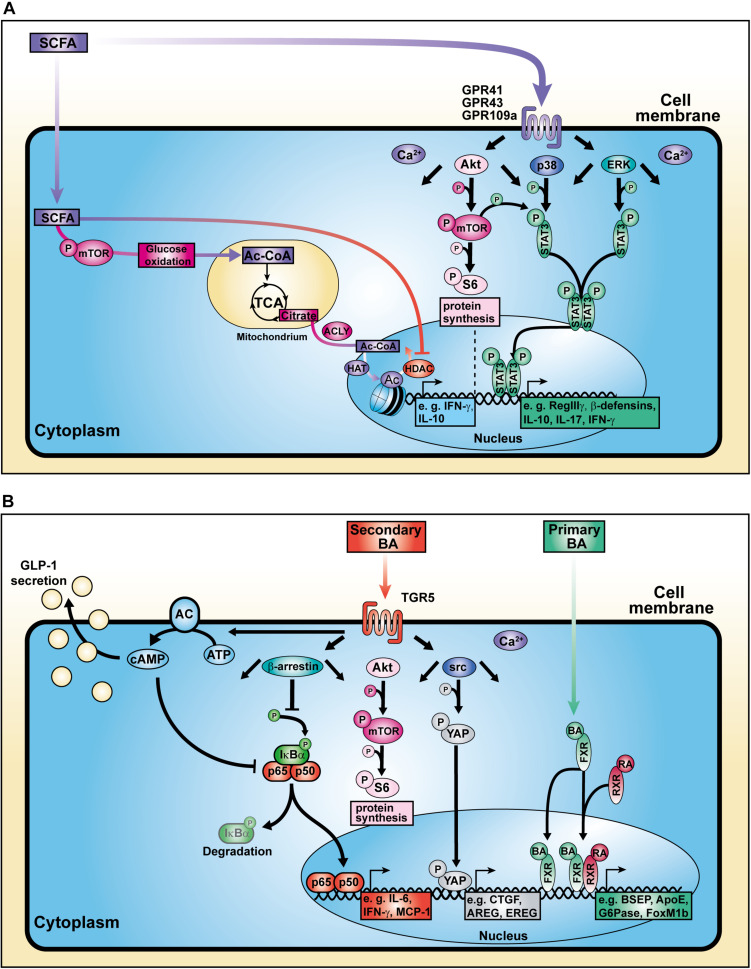
Cellular mechanisms of action of SCFAs and BAs. **(A)** SCFAs influence cell signaling, metabolic activity, and histone modification. **(B)** BAs bind to surface and nuclear receptors.

**FIGURE 3:**
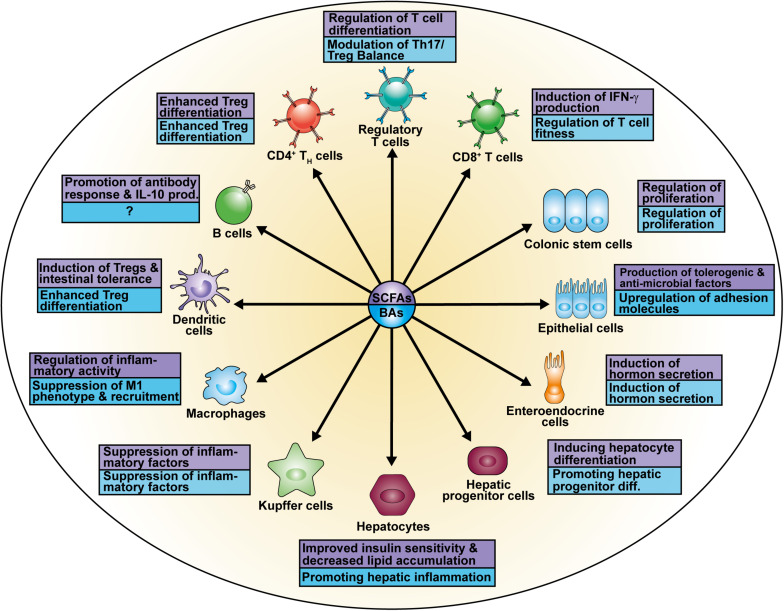
Cell types affected by SCFAs and BAs.

The research focus on SCFAs as a major class of bacterial-derived metabolites has revealed various of their modes of action as well as different cellular modifications (Figure 2A) depending on the respective cell type (Figure 3). The diffusible molecules have been shown to be agonists for eukaryotic GPRs which are involved in diverse signaling pathways. Previous studies have demonstrated that binding of acetate and propionate to GPR41 and GPR43 expressed on colonocytes induces p38 and ERK/MAPK activation contributing to the inflammatory response (Kim et al., 2013). Apart from colonocytes, enteroendocrine cells were shown to sense SCFAs via GPR41 and GPR43 (Nøhr et al., 2013). SCFA binding to GPR43 on regulatory T cells (Tregs) mediated protection against colitis in mice (Smith et al., 2013). Similarly, SCFA interaction with GPR109a on dendritic cells (DCs) promoted Treg differentiation and tolerance in the intestinal tissue (Singh et al., 2014).

Due to their small size, either passive diffusion across the cell membrane or active transport via sodium-coupled transporters enable SCFAs to enter the cytoplasma or even the nucleus of eukaryotic cells where they elicit a histone deacetylase (HDAC)-inhibitory activity of a distinct magnitude. A rather weak HDAC-inhibitory activity has been observed in experiments using acetate, while propionate and especially butyrate show stronger enzyme inhibition. SCFAs regulate the expression of genes associated with cell proliferation, differentiation, epithelial integrity, and immune response (Kim et al., 2014, 2016; Schilderink et al., 2016; Goverse et al., 2017; Luu et al., 2018). Recent studies have shown that not only enhanced histone H3 acetylation at the *Foxp3* locus but also increased acetylation of the Foxp3 protein itself can be modulated by butyrate, stabilizing the genetic integrity of Tregs (Arpaia et al., 2013; Furusawa et al., 2013).

Besides their HDAC-inhibitory properties, SCFAs are able to increase the activation of mammalian target of rapamycin (mTOR), a central regulator of cell growth and energy homeostasis (Sengupta et al., 2010). Consequently, mTOR-mediated enhancement of glycolysis contributes to the pool of acetyl-CoA. Balmer and colleagues showed that excess acetyl-CoA enters the tricarboxylic acid cycle (TCA) where it becomes converted into citrate. The pharmacologic inhibition of the ATP-citrate lyase (ACLY), an enzyme involved in the conversion of TCA-derived citrate into acetyl-CoA in the nucleus, strongly reduced the IFN-γ production in acetate-treated CD8^+^ memory T cells (Balmer et al., 2016). The nuclear acetyl-CoA served as a substrate for histone acetyltransferases (HATs) which facilitate the conjugation of acetyl groups to histones, thereby regulating gene expression and consequently the production of cytokines such as IL-10 and IFN-γ (Wellen et al., 2009; Zhao et al., 2016; Bantug et al., 2018; Luu et al., 2019). These data strongly support the concept of a metabolic-epigenetic crosstalk in which cellular metabolism-derived molecules serve as source for posttranslational modifications (PTMs) (Figure 2A).

As one of the most frequent PTMs, acetylation of proteins, such as histones, has been investigated intensively. Recent studies identified even longer alkyl motifs derived from SCFAs as substrates for histone modification (Kebede et al., 2017; Fellows et al., 2018). Kebede and colleagues described the propionylation and butyrylation of histone H3 as a novel mark of active chromatin in HeLa cells. In accordance with the concept that microbiota-derived metabolites act on the epigenetic state of host cells, it has been shown that antibiotic treatment reduces the microbial SCFA-mediated histone crotonylation in intestinal crypts (Fellows et al., 2018). These findings suggest a link between microbiota and epigenetic regulation opening the venue for investigating new PTMs based on microbial metabolites.

## SCFAs Impact on Intestinal Homeostasis and Inflammation

The gut is home to a diverse and dense bacterial community, a unique site of interaction between host and microbiota. Disturbance of this finely regulated balance was shown to be involved in inflammatory diseases such as inflammatory bowel disease (IBD) and colitis-associated carcinogenesis (CAC) (Kamada et al., 2013). Maintenance of the intestinal immune system requires an equilibrium between defense against pathogens as well as tolerance to commensals and food antigens. Therefore, various mechanisms are involved in regulating the immunological response, impacting on intestinal epithelial cells (IECs), induction of anti-inflammatory cells and suppression of inflammatory cells.

Recent studies have identified several effects of SCFAs on epithelial cells. SCFA administration was shown to stimulate retinoic acid (RA) production in the intestinal epithelium, a vitamin A derivate converted by aldehyde dehydrogenases, which is associated with signaling and expansion of peripheral Tregs (pTregs) in the context of a immunosuppressive response (Figure 3; Benson et al., 2007; Hill et al., 2008; Schilderink et al., 2016). Further, butyrate treatment of epithelial cells increased the production of IL-18 via a GPR109a-mediated mechanism which contributes to intestinal homeostasis and protects against colorectal carcinogenesis (Kalina et al., 2002; Zaki et al., 2010; Singh et al., 2014). Similarly, binding of SCFAs to GPR41 and GPR43 enhanced both expression of anti-microbial factors such as RegIIIγ and β-defensins in IEC by enhancing mTOR and STAT3 signaling, whereas mice deficient for the receptors suffered from impaired immune response against *C. rodentium* infection (Kim et al., 2013; Zhao et al., 2018). Besides regulation of anti-microbial molecules in epithelial cells, the increase in metabolic input and consequently acetyl-CoA upon SCFA or dietary fiber administration also regulates genes involved in plasma cell differentiation and IgA antibody production, important factors in maintaining gut homeostasis as well (Kim et al., 2016). The relevance of bacterial-derived SCFAs to gut homeostasis has been investigated in GF mice suffering from reduced mucosal integrity and IgA (Moreau et al., 1978; Duboc et al., 2013; Zeng et al., 2016).

SCFAs also mediate immunosuppression by either inducing IL-10 in different immune cells or repressing inflammatory macrophages in the lamina propria causing hyporesponsiveness to commensal bacteria (Hayashi et al., 2013; Sun et al., 2018). The importance of this aspect in IBD was highlighted by antibiotics-treated mice deficient for SCFAs and suffering from hyperresponsive macrophages and inflammation (Scott et al., 2018). A link between SCFA-mediated mTOR activation and IL-10 production in T cells and regulatory B cells (Bregs) has recently been shown by our lab investigating the subdominant microbiota-derived pentanoate (also known as valerate). Pentanoate enhanced glycolysis and consequently intracellular acetyl-CoA levels as HAT substrate, suggesting a regulation of the *Il10* locus by this mechanism (Luu et al., 2019).

The research on Tregs as crucial mediators of gut homeostasis and oral tolerance has identified acetate, propionate and butyrate as central molecules bridging the gap between commensals and the mucosal immune system. Their importance was emphasized by restoration of the colonic Treg population in GF mice, lacking both microbiota and commensal-derived metabolites, by SCFA supplementation (Smith et al., 2013). Different mechanisms underlying colonic Treg expansion have been proposed. Inhibition of HDACs leads to hyperacetylation at histone H3 and H4, particularly the acetylation in the promoter and CNS regions of the *Foxp3* locus which causes an increased expression of the Treg master regulator (Furusawa et al., 2013). Furthermore, enhanced acetylation of the Foxp3 protein itself upon butyrate treatment was shown to stabilize this transcription factor, protecting it from degradation (Arpaia et al., 2013). Apart from acting as HDAC inhibitors, Smith and colleagues described GPR43 to be exclusively expressed on colonic Tregs but not on those of other tissues, thereby pointing out the receptor-mediated Treg induction by SCFAs (Smith et al., 2013). Recent studies have identified several *Clostridium* strains among the commensal species, shown to facilitate colonic Treg maturation (Atarashi et al., 2011, 2013). Colonization of mice with 17 *Clostridium* strains producing SCFAs isolated from healthy humans resulted in a TGF-β-rich environment which supported Treg expansion and differentiation (Atarashi et al., 2013). Moreover, spore-forming *Clostridia* are involved in the fermentation of indigestible dietary fiber in the colon fueling the pool of SCFAs as key metabolites (Koh et al., 2016). In conclusion, these results demonstrate the complexity of microbiota-mediated regulation of gut homeostasis.

## SCFAs in Development of Colorectal Cancer and Stem Cell Renewal

Although the contribution of SCFAs to maintenance of gut homeostasis has been investigated extensively, there is an increasing body of evidence that commensal bacteria and bacterial metabolites have opposing roles in inflammatory responses and carcinogenesis depending on the cell type and the environment. Park and colleagues revealed that HDAC inhibition and mTOR activation rather than interaction with GPR43 is functionally important for the impact of SCFAs on T cells (Park et al., 2014). Further, they suggested that this main class of microbial metabolites boosts differentiation of naïve T cells into Th1 and Th17 cells during encounter with pathogens. Since these T cell subtypes were also described to be involved in colitis and inflammation-associated carcinogenesis in the colon, an involvement of SCFAs in colon carcinogenesis has been investigated recently (Grivennikov et al., 2010).

In a steady-state situation, butyrate is present in the mM range in the gut lumen and serves as the primary energy source for colonocytes (Flint et al., 2012). Butyrate’s HDAC-inhibitory properties have been pointed out by various studies (Park et al., 2014; Kespohl et al., 2017; Luu et al., 2018, 2019). Further, its impact on the cellular metabolism of colonocytes was investigated by the analysis of colonic tissue derived from GF mice. Colonocytes from these mice showed an energy-deprived state accompanied by reduced levels of enzymes involved in intermediary metabolism such as the TCA cycle, oxidative phosphorylation and ATP. Reconstitution with butyrate restored mitochondrial respiration in GF colonocytes (Donohoe et al., 2011). However, in the context of cancerous colonocytes, butyrate was shown to act paradoxically. While low-dose butyrate stimulates the proliferation of cancerous colonocytes not undergoing the Warburg effect in a low-glucose environment, comparably to non-cancerous ones, butyrate inhibits the proliferation of colonocytes utilizing the Warburg effect in a glucose-rich environment (Donohoe et al., 2012). The anti-proliferative effect of butyrate on glycolytic cells was attributed to histone hyperacetylation and changes in expression of genes involved in proliferation and apoptosis. High-dose butyrate caused histone hyperacetylation via its HDAC-inhibitory properties, whereas low-dose butyrate provided acetyl groups for HATs through its metabolization (Donohoe et al., 2012). These results emphasize a differential utilization of butyrate depending on the metabolic demand of the respective cell. In contrast to the potentially beneficial inhibition of cancerous colonocytes, the metabolization of butyrate by normal colonic epithelial cells was shown to mediate protection from its rather detrimental effect on colonic stem cells. Kaiko and colleagues exposed stem/progenitor cells *in vivo* to butyrate either by mucosal injury or application to zebrafish, naturally crypt-less host organisms, resulting in inhibited cell proliferation as well as impaired wound repair. These results suggested that the crypt structure anatomy might have co-evolved with metabolic pathways reacting to the microbiome (Kaiko et al., 2016). In contrast to the inhibitory effect of butyrate on colonic stem cells, high butyrate concentrations promoted differentiation of embryonic stem cells into hepatic progenitor cells (Ren et al., 2010). Interestingly, conversion of phytate into inositol-1,4,5-triposphate by commensals was identified as HDAC3 modulator countering the inhibition of epithelial growth by butyrate (Wu et al., 2020). In addition to that, butyrate is capable of promoting carcinogenesis in a genetic mouse model based on mutations in the *Apc* and the mismatch repair gene *Msh2* (*Apc*^*Min*/+^;*Msh2*^–/–^) (Belcheva et al., 2014). The authors showed an inflammation-independent contribution of butyrate to tumor development which was likely associated with an increased proliferation of epithelial stem cells and was reduced by feeding low-carbohydrate diet, linking the impact of microbiota and nutrition on tumorigenesis.

## SCFAs and BAs Impact on Liver Function, Inflammation and Carcinogenesis

The fermentation of dietary soluble fiber into SCFAs has been considered broadly as beneficial, promoting studies investigating the effect of various diets on host immunity and pathophysiology (Trompette et al., 2014; Tan et al., 2016; Zou et al., 2018). Although most of the interactions between diet, immune system and microbiota have been observed in the gut tissue, effects on organs in the periphery were described (Trompette et al., 2014; Haghikia et al., 2016). Recently, the profile of gut microbiota derived from feces of cirrhotic patients with hepatocellular carcinoma (HCC) showed an increase in *E. coli* pointing out that liver function is influenced by gut microbes (Gra̧t et al., 2016). It has been hypothesized that detection of the microbiota via surface receptors on hepatocytes such as TLR4 or TLR9 contributes to chronic liver injury, thus promoting cholestasis and HCC (Dapito et al., 2012; Cai et al., 2017). In contrast, TLR5 on hepatocytes mediated protection in a mouse model of high-fat-diet (HFD)-induced liver steatosis being important for bacterial clearance (Etienne-Mesmin et al., 2016). Emerging evidence has implicated that HFD enhances intestinal permeability, inflammation, and disease risk (Murphy et al., 2015). In mouse models, HFD induced alteration of the microbiota composition and reduced microbial diversity (Liu et al., 2021). Interestingly, clinical trials associated diet-induced obesity with decrease of microbial gene richness as well as increase of low-grade inflammation (Cotillard et al., 2013). Dysbiosis was further shown to cause reduction of intestinal integrity leading subsequently to bacterial translocation and endotoxemia (Cani et al., 2008; Dapito et al., 2012). Hence, HFD links diffusion of lipopolysaccharide from the gut to systemic inflammatory response as an exemplary factor involved in the gut-liver-axis (Cani et al., 2007, 2008; Laugerette et al., 2012).

A growing body of evidence suggests that SCFAs are key molecules of the gut-liver-axis with the capacity to either indirectly or directly impact on physiological liver function. On the one hand, they trigger the secretion of gut hormones such as GLP-1 by enteroendocrine cells improving glucose tolerance (Tolhurst et al., 2012). These effects have been attributed to GPR41 and GPR43 signaling in L cells which showed increased surface expression of both receptors. Ablation of these beneficial effects on liver function was observed in *Gpr41-*/ *Gpr43-*deficient mice (Tolhurst et al., 2012; Shimizu et al., 2019). On the other hand, the portal circulation enables the direction of gut-derived SCFAs toward the liver. Based on experiments with physiological amounts of isotope-labeled SCFAs, den Besten and colleagues demonstrated that 62% of the infused propionate in the murine cecum was involved in whole body glucose production, accounting for 69% of total glucose synthesis (den Besten et al., 2013). Consistently, hepatocyte-like cells in a coculture system with epithelial cells were treated with propionate and showed increased glycogen synthesis as well as storage (El Hage et al., 2020). Further, oral administration of HFD supplemented with acetate and propionate in different ratios was associated with increased insulin sensitivity and reduced triglyceride content in the liver (Weitkunat et al., 2016). Mice fed either HAc or HPr showed lower blood glucose levels 240 min after glucose administration as compared to HFD feeding. At the same time, decreased levels of plasma insulin were detected in the HAc and HPr groups. These results highlighted a more efficient glucose uptake with a lower demand for insulin upon SCFA treatment (Weitkunat et al., 2017). The beneficial effects on insulin resistance might be attributed to the previous observation that SCFAs promote GLP-1 secretion (Tolhurst et al., 2012).

It has been found that SCFAs are able to stimulate hormone secretion by enteroendocrine cells but their effect on hepatic responsiveness was investigated to lesser extent. A recent study showed similar levels of GLP-1 in the serum of NAFLD patients and healthy controls but detected a downregulation of the hepatic GLP-1 receptor (GLP-1R) in NAFLD patients. In a mouse model of NAFLD, administration of butyrate reversed the reduction of GLP-1R and led to upregulation of hepatic AMPK phosphorylation and insulin receptor expression in treated mice. Moreover, increase in GLP-1R expression levels in HepG2 cells was mediated by butyrate’s HDAC-inhibitory activity, acting indepently of GPR43 and GPR109a (Zhou et al., 2018). Interestingly, binding of propionate to GPR43 on hepatic tumor cells inhibited their growth (Bindels et al., 2012). Early studies investigating the effect of butyrate on a myeloid subset of hepatic cells, Kupffer cells, showed a significant decrease in TNF-α as well as an increase in prostaglandin E2 (PEG2) production (Perez R. et al., 1998; Perez R.V. et al., 1998). These observations highlighted a potential role of butyrate in Kupffer cell immunoregulation which might protect from HCC by alleviating inflammatory responses as prerequisite.

In addition, the role of SCFAs, BAs and diet in HCC development was investigated resulting in opposing observations. Singh and colleagues have found that the addition of the soluble fiber inulin to the diet induced HCC in a microbiota-dependent manner in dysbiotic mice but not in germ-free or antibiotics-treated mice (Singh et al., 2018). Moreover, inulin-enriched HFD promoted dysbiosis and HCC in WT mice which was associated with liver inflammation, neutrophil influx and cholestasis (Figure 1). These pathologies were ameliorated by either depleting butyrate-producing bacteria or excluding soluble fiber from diet as source of SCFA generation. Of note, inhibition of the enterohepatic recycling of BAs reduced liver carcinogenesis suggesting their involvement in hepatic inflammation (Singh et al., 2018). The nourishment of mice with fermentable dietary fiber guar gum altered the gut microbiota composition and elevated the bile acid levels in the liver (Janssen et al., 2017). Although diet-induced obesity was reduced, guar gum-related BA levels enhanced liver inflammation and fibrosis. Consistently, administration of taurocholic acid led also to hepatic inflammation which could be reduced by antibiotics treatment (Janssen et al., 2017). In agreement with these findings, analysis of gut microbiome from stool of non-alcoholic fatty liver disease (NAFLD) patients, who suffer from adipokine dysregulation, insulin resistance and fat accumulation in the liver, revealed elevated levels of propionate and BAs (Lee et al., 2020).

While various mechanisms have been proposed for SCFA activity, the effects of BAs on liver injury remain controversial. Treatment of murine and human hepatocytes with pathophysiologic levels of BAs induced the expression of pro-inflammatory cytokines which recruit neutrophils to the hepatic tissue in a CCL2-dependent manner. In addition, TLR9 in hepatocytes was identified as an important mediator of BA-induced liver inflammation (Cai et al., 2017).

Yamada and colleagues demonstrated that secondary BAs promote HCC development in a model of non-alcoholic steatohepatitis (NASH), a progressive form of NAFLD characterized by liver inflammation and fibrosis with the potential to develop into HCC (Yamada et al., 2018). The group fed a new class of steatohepatitis-inducing high fat diet (STHD-01), inducing NASH within 9 weeks post administration consequently progressing into HCC after 41 weeks in WT mice. Accumulation of both cholesterol and BAs in liver and feces were observed after STHD feeding. Interestingly, antibiotics treatment reduced the accumulation of secondary BAs suggesting an impact on bacterial conversion of primary BAs. The group hypothesized that secondary BA-induced mTOR activation in the liver might be responsible for hepatic carcinogenesis in NASH. Another study depicted a connection between circulating BAs and mTOR signaling via the Takeda G protein-coupled receptor 5 (TGR5), emphasizing the role of BA receptors in modulating cellular processes (Figure 2B; Zhai et al., 2018).

## BA Receptors Have an Ambiguous Role in Shaping the Microbiome, Liver Function and Inflammatory Response

Researchers have focused on the regulation of BA and host metabolism mediated by the farnesoid X receptor (FXR) and TGR5 (Figure 1). The FXR is a nuclear receptor with the highest expression in liver and ileum functioning as a transcription factor which regulates various target genes either as monomer or upon dimerization with the retinoid X receptor (RXR) and subsequent promoter binding (Figure 2B; Kassam et al., 2003; Lefebvre et al., 2009; Teodoro et al., 2011). The target genes are related to BA, lipoprotein and glucose metabolism (e.g., BSEP, ApoE, G6Pase) but also to liver regeneration as demonstrated for the expression of transcription factor FoxM1b (Huang et al., 2006; Wang et al., 2008). With respect to liver regeneration, BA signaling was shown to promote stem cell differentiation toward hepatocytes (Sawitza et al., 2015).

The generation of *Fxr*-deficient mice was a prerequisite for mechanistic studies which showed partially contradictory results, attributed to differences in diet, genetic background as well as microbiota changes among animal facilities. These mice fed chow diet were susceptible to hyperglycemia and hypercholesterolemia (Lambert et al., 2003; Ma et al., 2006). On the one hand, *Fxr*-deficient mice on *Ldlr*^–/–^ background were protected against HFD-induced obesity and atherosclerosis (Zhang et al., 2012). On the other hand, *Fxr*-deficient mice on *Apoe*^–/–^ background showed elevated atherosclerosis scores (Hanniman et al., 2005). However, in the context of HFD and genetically obese backgrounds (ob/ob), *Fxr*-deficient mice prevalently showed beneficial effects with regards to glucose homeostasis and obesity (Prawitt et al., 2011; Zhang et al., 2012; Parséus et al., 2017). Similar discrepancies were observed in studies investigating the effect of intestinal and hepatic *Fxr*-deficiency on liver steatosis. FXR expression in the gut was shown to mediate HFD-induced NAFLD, whereas liver-specific FXR activity protected against hepatic steatosis (Li et al., 2013; Jiang et al., 2015; Schmitt et al., 2015).

The gut microbiota has been identified as another crucial factor impacting FXR signaling. Sayin and colleagues revealed that the primary BA tauro-β-mauricholic (TβMCA) can be metabolized by gut bacteria (Sayin et al., 2013). Hence, reduction of the natural FXR antagonist improved FXR signaling in mice. Additionally, microbial activity provides secondary BAs as TGR5 ligands by conversion of primary ones (Kuipers et al., 2014). Experimental approaches with gnotobiotic animals revealed that microbiota influences diet-induced obesity in a FXR-dependent manner (Li et al., 2013; Jiang et al., 2015). Further, transfer of microbiota derived from HFD-fed *Fxr*-deficient into GF mice inhibited weight gain compared to bacterial colonization from WT mice (Parséus et al., 2017). *Fxr*-deficient mice on HFD not only showed enhanced levels of the primary BAs βMCA and TβMCA. At the same time, BA profiles of GF *Fxr*-deficient and WT animals were comparable. These findings suggest that the altered microbiota has reduced conversion of primary to secondary BAs due to FXR deletion.

While gut bacteria contribute to the pool of available BAs, vice versa, these are able to shape the microbial composition by either supporting the growth of BA-metabolizing bacteria or growth inhibition of bile sensitive bacteria. Early studies have observed that blockade of bile flow into the gut as result of a biliary obstruction led to bacterial translocation (Clements et al., 1996). Interestingly, experiments in rats showed that bacterial expansion can be reduced by oral bile acid treatment (Lorenzo-Zúñiga et al., 2003). Besides the intrinsic bactericidal properties of BAs, stimulation of FXR induces the production of antimicrobial molecules by immune cells, additionally shaping the microbial colonization (Inagaki et al., 2006).

The membrane-bound G protein-coupled receptor TGR5 is ubiquitously expressed in various tissues such as intestine, liver and gallbladder (Cipriani et al., 2011; Bidault-Jourdainne et al., 2021). In contrast to FXR, TGR5 mainly binds secondary BAs. Therefore, *Tgr5*-deficient mice have served as models to investigate its impact on BA and microbial composition. Although breeding of these mice resulted in healthy offspring, a reduction of the bile acid pool suggested a role of TGR5 in BA homeostasis (Maruyama et al., 2006).

In a model of diet-induced obesity, Thomas and colleagues found that TGR5 signaling leads to glucagon-like peptide-1 (GLP-1) secretion by enteroendocrine cells (Figure 2B; Thomas et al., 2009). Thereby, improvement of both pancreatic and liver function as well as tolerance of glucose were observed in obese mice. In addition to that, TGR5 targeting with the specific agonist INT-777 inhibited hepatosteatosis, offering a treatment option for metabolic diseases. Moreover, TGR5 stimulation is involved in the expression of junctional adhesion molecule A (JAM-A) by biliary epithelial cells (Merlen et al., 2020). While JAM-A was downregulated as well as hypophosphorylated in BA ducts and gallbladder from *Tgr5*-deficient mice, administration of a specific TGR5 agonist in WT mice stabilized the adhesion molecule via JAM-A Ser28 phosphorylation. Additionally, TGR5-agonist-treated mice were less susceptible to choleostasis-induced liver damage due to reduced bile leakage, in contrast to JAM-A-KO mice. Hence, hepatic TGR5 signaling mediates liver protection. Interestingly, also in the context of intestinal inflammation, TGR5 deletion was associated with increased intestinal permeability leading to higher severity during colitis (Cipriani et al., 2011). Additionally, TGR5 activation was shown to activate intestinal stem cells inducing Src/YAP-driven regeneration in response to tissue injury (Sorrentino et al., 2020).

Similarly, it was shown that TGR5 deletion in a model of alcohol-induced liver disease causes even greater liver damage as a result of steatosis and inflammation (Spatz et al., 2021). This phenotype was related to enhanced recruitment of inflammatory macrophages to the liver. Furthermore, deficiency in the BA receptor resulted in dysbiosis as demonstrated by microbiota transfer from *Tgr5*-deficient mice into their WT counterparts, worsening alcohol-mediated hepatic inflammation. Of note, the pool of secondary BAs was reduced in these animals attributed to a lower abundance of bacterial genes related to BA transformation. The importance of BA transformation was further demonstrated in the work by Ma and colleagues which showed that the conversion of primary into secondary BAs by *Clostridium* species repressed production of CXCL16 in liver sinusoidal endothelial cells. Antibiotic depletion of BA-transforming bacteria increased the levels of primary BAs and CXCL16 production. Subsequent recruitment of natural killer T cells controlled growth of liver cancer (Ma et al., 2018).

Recently, investigations of inflammatory macrophages in chronic liver disease pointed out that TGR5 expression was reduced in liver samples from humans and mice suffering from NASH (Shi et al., 2020). The group described that macrophages derived from *Tgr5*-deficient were prone to M1 polarization accompanied by pro-inflammatory cytokine production. Mechanistically, TGR5 inhibits the NLRP3 inflammasome activation as well as caspase-1 cleavage, protecting against liver steatosis. Data supporting the contribution of TGR5 deletion to inflammation revealed that *Tgr5*-deficient macrophages and Kupffer cell enhanced the expression of pro-inflammatory factors such as IL-6 and MCP-1 in response to LPS. This phenotype was associated with reduced β-arrestin2-dependent suppression of the NF-κB pathway (Figure 2B; Witherow et al., 2004; Wang et al., 2011). Importantly, it was pointed out that the inflammatory activity of Kupffer cells promotes the progression of HCC (Maeda et al., 2005; He and Karin, 2011).

While the mentioned studies have focused on inflammatory myeloid cells in the onset of BA-dependent liver disease, a recent study has identified a role of BAs in inducing immunosuppressive Treg response via DCs. The secondary BA 3β-hydroxydeoxycholic acid (isoDCA) was shown to reduce the immunostimulatory activity of DCs and to enhance Treg differentiation (Campbell et al., 2020b). Genetic deletion of FXR in DCs mimicked the effects of isoDCA administration suggesting an antagonistic mechanism involved in Treg generation. Furthermore, the design of a bacterial consortium comprised of isoDCA-producing *Bacteroides* strains induced the differentiation of colonic RORγt^+^ Tregs. Another study revealed that the Treg-intrinsic BA receptor VDR contributes to the pool of extrathymic Tregs which elicited anti-inflammatory response during colitis (Song et al., 2020). Interestingly, Hang and colleagues (2019) suggested another mechanism by which the BAs 3-oxolithocholic acid (3-OxoLCA) and isoallolithocholic acid (isoalloLCA) act directly on T cells. Binding of 3-OxoLCA to RORγt inhibited development of TH17 cells, whereas induction of mitochondrial reactive oxygen species by isoalloLCA enhanced Treg differentiation. Thereby, BAs are able to modulate the Th17/Treg balance which is an example of how they contribute to immunoregulatory mechanisms of gut homeostasis, rather than acting pro-inflammatory, as part of the gut-liver-axis.

## Conclusion and Future Directions

Various studies have highlighted microbiota-derived SCFAs and BAs as factors impacting host physiology, development of diseases and outcome of treatment strategies. The identification of microbial metabolites and their respective modes of action might be crucial for the development of new therapeutic approaches and identification of biomarkers.

Especially with respect to SCFAs, different mechanisms were identified by which these small aliphatic molecules influence cellular signaling, metabolism and epigenetics (Kim et al., 2013, 2016; Luu et al., 2018). However, most studies have investigated these independently, not covering the potential crosstalk between the pathways. Recently, a connection between the induction of the mTOR pathway and the provision of acetyl-CoA as substrate for HAT-mediated histone acetylation in lymphocytes was pointed out (Luu et al., 2019; Qiu et al., 2019). Additionally, Schulthess and co-workers described a link between butyrate-mediated inhibition of HDAC3 and the decrease in mTOR activity by macrophages (Schulthess et al., 2019). These results emphasize a bidirectional connection between the epigenetic and metabolic pathways and that more research should be invested into unraveling pathway inter-connectivity to understand cell-specific regulatory networks.

Considering the utilization of acetate as source for PTMs, it is conceivable that even longer SCFA-derived histone alkylations will be identified as new biomarkers (Kebede et al., 2017; Fellows et al., 2018). More experimental evidence is needed to evaluate their biological role in transcriptional regulation of cells such as colonocytes and intestinal immune cells and their potential as therapeutic or diagnostic markers.

Although SCFAs are considered for therapeutic administration, studies have reported contradictory observations with regards to the effects of SCFAs. Their effects have been described as both beneficial and adverse depending on the disease model (Smith et al., 2013; Singh et al., 2014, 2018; Trompette et al., 2014; Kim et al., 2016; Kespohl et al., 2017). Although the research on these small molecules was focused on their immunosuppressive capacities, recent data propose a role in inflammatory responses (Smith et al., 2013; Trompette et al., 2014; Kespohl et al., 2017; Luu et al., 2018, 2019; Bachem et al., 2019). For instance, it has been reported that SCFAs are able to boost Th17 differentiation as benefical effect upon pathogen encounter but similar mechanisms were demonstrated to repress Th17 cells in a mouse model of autoimmune encephalomyelitis (Park et al., 2014; Luu et al., 2019). We have recently shown that SCFAs are also capable of promoting the cytotoxic phenotype of tumor-specific CD8^+^ T cells and chimeric antigen receptor (CAR) T cells, thereby enhancing their anti-tumor activity (Luu et al., 2021). These findings suggest that the impact of microbial metabolites is highly context-dependent. Hence, future work should investigate both their immunostimulatory and immunosuppressive capacities for a comprehensive analysis.

Likewise, studies have reported contradictory observations with regards to the effects of BAs in genetically modified animals and different disease models which need further investigation (Hanniman et al., 2005; Zhang et al., 2012; Li et al., 2013; Schmitt et al., 2015; Parséus et al., 2017).

As an example, the work with *Fxr*-deficient animals has identified the genetic background as crucial factor influencing the experimental outcome. *Fxr*-deficient mice on *Ldlr*^–/–^ and ob/ob background were protected against HFD-induced obesity and atherosclerosis in contrast to mice on *Apoe*-/- background (Hanniman et al., 2005; Ma et al., 2006; Zhang et al., 2012). Even work on organ-specific FXR-deletion in the liver and intestinal tissue showed conflicting results underlining the need for a systematic comparison between these models (Li et al., 2013; Schmitt et al., 2015). The development of next generation *in vivo* tools might comprise tissue- or cell-specific FXR-deletion combined with *Ldlr*- or *Apoe*-deficiency in the same tissue rather than systemic knockouts, thereby reducing incidental effects.

Moreover, the reviewed studies were dependent on administration of fiber-rich diet or HFD to either shape the microbial composition or to stimulate inflammatory response. Due to a wide range of commercially available diets and supplements, the exact composition might differ between the studies (Pellizzon, 2016). Standardization of diet composition for *in vivo* experiments could help to unify newly developed models.

With regards to standardization, the microbial composition is another crucial aspect impacting on disease outcome. A prominent example is the *Il10*-deficient mouse model for spontaneous colitis. Different groups have reported varying histopathology scores depending on the SPF condition in their respective animal facility and the colonization with commensal strains (Burich et al., 2001; Balish and Warner, 2002; Schultz et al., 2002; Keubler et al., 2015). Moreover, Ivanov and colleagues (2009) identified segmented filamentous bacteria (SFB) in the murine gut as inducer of intestinal Th17 cells which were demonstrated to promote central nervous system autoimmmunity (Luu et al., 2019). Of note, SFB were present in the gut of WT mice derived from Taconic Farms, while there were not detected in those raised at Jackson Laboratory (Ivanov et al., 2009). Therefore, not only uniformity of bacterial colonization in WT mice but also of its variations in genetically modified strains needs to be considered to assure reproducible results and will additionally improve the monitoring of changes within the microbiota composition over the course of the experiment.

Although the impact of BAs on various immune cell types has been investigated, their effect on B cells has not been described yet. Recent studies demonstrated that microbiota-derived serotonine-derivates and SCFAs can act as aryl-hydrocarbon receptor ligands or metabolic enhancer in regulatory B cells (Bregs), respectively (Rosser et al., 2020). Also the finding that BAs impact on intestinal Treg differentiation underlines their immunosuppressive capacities (Hang et al., 2019; Song et al., 2020). As BAs are capable of inducing either tolerogenic or inflammatory responses, it remains to be clarified whether B cells are prone to become Bregs, antigen-presenting or plasma cells upon BA treatment. Also the interaction between BAs and CD8^+^ T cells has been investigated insufficiently. Both the involvement of CD8^+^ T cells in controlling the BA synthesis by inducing cholangitis and the effect of FXR deletion on T cell fitness were described previously (Glaser et al., 2019; Campbell et al., 2020a). In addition, a recent study has revealed accumulation of CXCR6^+^ auto-aggressive CD8^+^ T cells in the liver of mice and patients suffering from NASH (Dudek et al., 2021). Yet, the underlying mechanisms linked to BAs as well as the assessment of *Fxr*- and *Tgr5*-deficient CD8^+^ T cell in the context of NASH or HCC remain to be subject of future work.

Finally, there are gaps in our knowledge regarding the inter-connectivity between SCFA and BA biology. Although many mechanistic insights were provided by work on either SCFAs or BAs, their simultaneous impact on the same pathways, synergistic or opposing effects have not been elucidated in depth. For instance, the activity of SCFAs as GPR41/GPR43 agonists might influence the BA-induced signaling via TGR5. Moreover, limited data is available on the interaction between SCFA producers, BA-sensitive and BA-transforming bacteria. A first attempt of analyzing the interaction between SCFAs and BAs was analyzed by Sheng and colleagues. The group has demonstrated that lack of butyrate-producing bacteria enhanced hepatitis in *Fxr*-deficient mice fed a western diet, while administration of butyrate reversed inflammation caused by the *Fxr*-deficiency-derived BA dysregulation (Sheng et al., 2017). This work highlighted the potential of the joint expertise from the SCFA and BA biology field which might enable future research to fill the gaps within our knowledge with respect to the complex inter-kingdom crosstalk between commensals and eukaryotic cells.

## Author Contributions

Both authors listed have made a substantial, direct and intellectual contribution to the work, and approved it for publication.

## Conflict of Interest

The authors declare that the research was conducted in the absence of any commercial or financial relationships that could be construed as a potential conflict of interest.

## Publisher’s Note

All claims expressed in this article are solely those of the authors and do not necessarily represent those of their affiliated organizations, or those of the publisher, the editors and the reviewers. Any product that may be evaluated in this article, or claim that may be made by its manufacturer, is not guaranteed or endorsed by the publisher.
